# JsrNet: A Joint Sampling–Reconstruction Framework for Distributed Compressive Video Sensing

**DOI:** 10.3390/s20010206

**Published:** 2019-12-30

**Authors:** Can Chen, Yutong Wu, Chao Zhou, Dengyin Zhang

**Affiliations:** 1 College of Telecommunications and Information Engineering, Nanjing University of Posts and Telecommunications, Nanjing 210003, China; chencan15@126.com (C.C.); zhouchaogl@163.com (C.Z.); 2 College of Information Technology, Shanghai Ocean University, Shanghai 201306, China; wuyutong20000801@126.com; 3 College of Internet of Things, Nanjing University of Posts and Telecommunications, Nanjing 210003, China; 4 Jiangsu Key Laboratory of Broadband Wireless Communication and Internet of Things, Nanjing University of Posts and Telecommunications, Nanjing 210003, China

**Keywords:** distributed compressive video sensing, deep convolutional neural networks, video signal processing

## Abstract

Huge video data has posed great challenges on computing power and storage space, triggering the emergence of distributed compressive video sensing (DCVS). Hardware-friendly characteristics of this technique have consolidated its position as one of the most powerful architectures in source-limited scenarios, namely, wireless video sensor networks (WVSNs). Recently, deep convolutional neural networks (DCNNs) are successfully applied in DCVS because traditional optimization-based methods are computationally elaborate and hard to meet the requirements of real-time applications. In this paper, we propose a joint sampling–reconstruction framework for DCVS, named “JsrNet”. JsrNet utilizes the whole group of frames as the reference to reconstruct each frame, regardless of key frames and non-key frames, while the existing frameworks only utilize key frames as the reference to reconstruct non-key frames. Moreover, different from the existing frameworks which only focus on exploiting complementary information between frames in joint reconstruction, JsrNet also applies this conception in joint sampling by adopting learnable convolutions to sample multiple frames jointly and simultaneously in an encoder. JsrNet fully exploits spatial–temporal correlation in both sampling and reconstruction, and achieves a competitive performance in both the quality of reconstruction and computational complexity, making it a promising candidate in source-limited, real-time scenarios.

## 1. Introduction

Compressive sensing (CS) [1,2] is a powerful framework for signal acquisition and processing. By adopting a measurement matrix, CS integrates sampling and compression, making it desirable in many applications such as magnetic resonance imaging (MRI) [3] and cognitive radio communication [4]. CS states that if the measurement matrix satisfies the restricted isometry property (RIP), we can recover one sparse or compressible signal from fewer measurements than that suggested by the Nyquist theory [5]. Frame-based sampling [6,7] is impractical due to limited storage space. To overcome this problem, Lu [8] proposed block-based CS that reduced much of the implementation costs.

Over the past decade, CS has been successfully applied in video signal processing because compared to still images, video signals contain more spatial and temporal redundancies which can be further exploited. One of the most powerful architectures in video CS reconstruction in the literature is distributed compressive video sensing (DCVS), which is desirable in source-limited scenarios because of its hardware-friendly characteristics. In DCVS, the first frame of a given group of frames is classified as the key frame and the remaining frames are classified as non-key frames. In an encoder, each frame is sampled independently; in a decoder, key frames are reconstructed independently and served as references in the recovery of non-key frames. A large number of algorithms have been proposed for DCVS, which focus on how to further exploit spatial–temporal correlation in decoders to improve reconstruction performance. Inspired by motion estimation (ME) and motion compensation (MC), the multi-hypothesis (MH) prediction algorithm [9] utilizes a combination of blocks to generate a prediction for the target block. Combining MH and residual reconstruction [10], the MH-BCS-SPL algorithm [11] yields state-of-the-art results for DCVS. Further improvements based on MH are proposed in [12,13]. Zhao [14] proposed a reweighted residual sparsity (RRS) model which not only takes full advantage of spatial correlation of videos to produce good initial recoveries, but also utilizes temporal correlation between frames to further enhance the reconstruction quality. To enhance the robustness of MH prediction, Chen [15] proposed a reweighted Tikhonov regularization which considers the impact of each hypothesis. Although these methods can yield competitive reconstruction quality, they are time-consuming and do not easily meet the requirements of real-time applications. Thus, MH-BCS-SPL is commonly adopted in DCVS for its acceptable reconstruction performance and low computational complexity [16,17,18].

Iterative optimization-based methods used in traditional DCVS are computationally elaborate and do not easily meet the requirements of real-time applications. Fortunately, as deep convolutional neural networks (DCNNs) have shown great potential in solving computer vision tasks, such as classification and object detection, applying DCNN to solve CS problem has attracted considerable attention. Different from traditional approaches, DCNN-based approaches utilize deep learning techniques to directly recover the original signal from the measurement vector, achieving a better trade-off between reconstruction quality and computational complexity. A stacked denoising autoencoder (SDA) [19] was first proposed to efficiently estimate a signal. DeepInverse [20] was first proposed to utilize a DCNN to learn inverse transformation. Inspired from the denoising-based approximate message passing (D-AMP) algorithm [7], Metzler [21] developed Learned D-AMP (LDAMP), which unrolls D-AMP algorithm into a novel neural network architecture. Reconnet [22] first reconstructs each block using a DCNN architecture and assembles reconstructed blocks to feed into an off-the-shelf denoiser. In Deepcodec [23], the sensing process of images is non-linear and learned from the training data. Recently, several video frameworks were proposed. Combining DCNNs and long short-term memory (LSTM) networks, CSVideoNet [24] achieves a promising performance in DCVS. Blocking artifacts were introduced in these methods because they neglect edge continuity between blocks. To reduce blocking artifacts, instead of utilizing post-processing [22], a novel network in which all measurements of blocks from one image are used simultaneously to reconstruct the full image was proposed in [25]. A multi-frame quality enhancement (MFQE) [26] approach based on LSTM networks was proposed, which enhances the quality of low-quality frames by using their neighboring high-quality frames.

The promise of the existing DCNN-based frameworks has been offset by two problems. First, the existing frameworks only utilize key frames as the reference to reconstruct non-key frames. Secondly, the conception of exploiting complementary information between frames is only applied in joint reconstruction. To address these problems, we propose a joint sampling–reconstruction framework for DCVS, named “JsrNet”. The main contributions of our work are three-fold:

1. JsrNet utilizes the whole group of frames as the reference to reconstruct each frame, regardless of key frames and non-key frames.
2. JsrNet not only applies the conception of exploiting complementary information between frames in joint reconstruction, but also in joint sampling by adopting learnable convolutions to sample multiple frames jointly and simultaneously in an encoder.
3. JsrNet exploits spatial–temporal correlation in both sampling and reconstruction, and achieves a competitive performance on both the quality of reconstruction and computational complexity, making it a promising candidate in source-limited, real-time scenarios.

The remainder of this paper is organized as follows. In Section 2, we review the backgrounds of our work. Section 3 introduces a detailed description of the proposed JsrNet. In Section 4, we provide the experimental results. Conclusions are drawn in Section 5.

## 2. Backgrounds

### 2.1. Preliminary of CS Theory

CS theory states that we can measure a signal $x\in R^{n\times 1}$ with a sub-Nyquist rate through a measurement matrix $\varphi \in R^{m\times n}$:

(1) y=φx,

where $y\in R^{m\times 1}$ denotes the measurements vector and SR=m/n denotes the sampling rate. In block-based CS, *n* is equal to $B^{2}$, where *B* denotes the block size. Since m≪n, the recovery of *x* from *y* is ill-posed. Regularized iterative algorithms [7,27] have become the standard approach to this ill-posed inverse problem in the past few decades:

(2) $$\underset{x}{\arg\min}\frac{1}{2}{\left\|y-\varphi x\right\|}_{2}^{2}+\lambda R(x),$$

where λ is a non-negative constant and *R*(*x*) represents some priors about the signal structure, such as sparse priors [28,29] and low-rank priors [30,31]. These methods suffer from high computational complexity and parameter-tuning issues. Due to the powerful learning capability of deep networks, deep learning-based algorithms [19,20,21,22,23,24,25,32] have successfully shown great potential in solving this inverse problem.

### 2.2. Unsupervised Learning

Both supervised learning and unsupervised learning have been successfully applied in image CS frameworks; however, we highlight the need for using unsupervised learning to find and represent structure in video CS frameworks because videos contain a large amount of spatial and temporal redundancies which makes them particularly suitable for building unsupervised learning models. This is consistent with one of the motivations of our work that we aim to apply the conception of exploiting complementary information between frames in joint sampling.

Given a *T*-length group of pictures $\left\{x_{1},\ldots ,x_{T}\right\}$, we use mean square error (MSE) as the loss function which favors high peak signal-to-noise ratio (PSNR):

(3) $$L(\Theta )=\frac{1}{2T}\sum_{i=1}^{T}{\left\|F(x_{i};\Theta )-x_{i}\right\|}_{2}^{2},$$

where Θ represents the parameters in the designed network and $F(x_{i};\Theta )$ denotes the output of the network. One advantage of these algorithms is low computational complexity because signals are reconstructed by feeding to a single forward model, instead of optimizing iteratively.

## 3. The Proposed JsrNet

**Figure 1:**
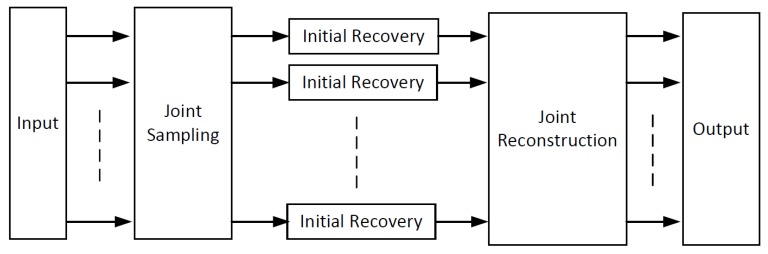
Overview architecture of JsrNet.

In this section, we propose a joint sampling–reconstruction framework for DCVS, named “JsrNet”. JsrNet measures signals in a block-based manner, but reconstructs signals in a frame-based manner. Figure 1 shows the overview architecture of JsrNet which contains three modules: (1) a convolutional neural network (CNN) for joint sampling, in which multiple frames are sampled jointly and simultaneously by using learnable convolutions in a block-based manner; (2) a spatial DCNN for initial recovery, in which all measurements of blocks from one image are used simultaneously to output the intermediate reconstructed image; and (3) a temporal DCNN for joint reconstruction, in which each frame is reconstructed by exploiting temporal correlation within the whole group of frames. These three modules consist of an integrated end-to-end model whose parameters are jointly trained.

### 3.1. CNN for Joint Sampling

**Figure 2:**
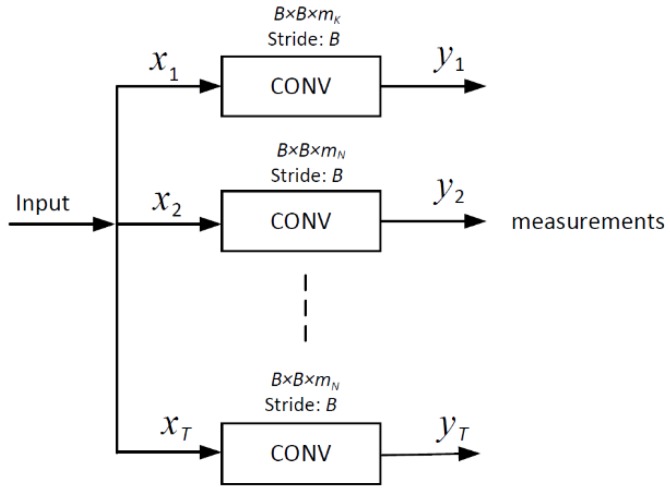
CNN for joint sampling. In a *T*-length group of pictures, the key frame $x_{1}$ and the remaining non-key frames $\left\{x_{2},\ldots ,x_{T}\right\}$ go through specific convolution layers to generate corresponding measurements $y_{i=1\ldots T}$.

Different from traditional approaches which commonly utilize the random Gaussian matrix [22] as the measurement matrix, we use a convolutional layer [33] in which parameters only depend on the size and number of convolution kernels to mimic the sampling operation. Figure 2 shows the structure of the encoder for joint sampling. First, video sequences are divided into several *T*-length groups of frames, in which a key frame $x_{1}$ is followed by some non-key frames $\left\{x_{2},\ldots ,x_{T}\right\}$. Each frame goes through a specific convolution layer in which rectified linear units (ReLU) activation [34] was removed to obtain measurements in a block-based manner. High sampling rates, $SR_{K}=m_{K}/n$, are allocated to key frames, whereas relatively low sampling rates, $SR_{N}=m_{N}/n$, are allocated to non-key frames. During the training process, the sampling of multiple frames is jointly optimized, fully exploiting spatial–temporal correlation in the encoder. Different from the existing frameworks which only focus on exploiting complementary information between frames in joint reconstruction, JsrNet also applies this conception in joint sampling by adopting learnable convolutions to sample multiple frames jointly and simultaneously in the encoder.

### 3.2. Spatial DCNN for Initial Recovery

**Figure 3:**
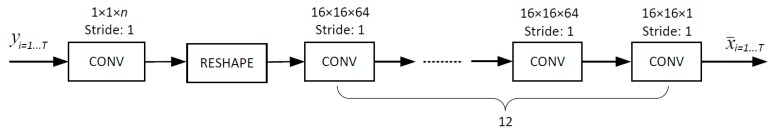
Spatial DCNN for initial recovery. Each intermediate reconstruction ${\bar{x}}_{i=1\ldots T}$ is recovered from corresponding measurements $y_{i=1\ldots T}$ through its corresponding spatial DCNN.

In this subsection, we design a spatial DCNN for the initial recovery of each frame which is shown in Figure 3. Inspired by [25] which effectively removes the blocking artifacts, all measurements of blocks from one image are used simultaneously to reconstruct the full image. Different from typical DCNNs used for classification and segmentation, we remove the pooling layer which can cause information loss. We first use a convolutional layer which uses *n* convolution kernels of size 1 × 1 with stride 1 and a reshape layer to transform the measurements to the feature map which has the same dimension as the final reconstructed frame. Then, we stack 12 convolutional layers to obtain the intermediate reconstruction ${\bar{x}}_{i=1\ldots T}$. All the convolutional layers are followed by ReLU activation, except the final layer, and each frame has its corresponding spatial DCNN, instead of a universal one.

### 3.3. Temporal DCNN for Joint Reconstruction

**Figure 4:**
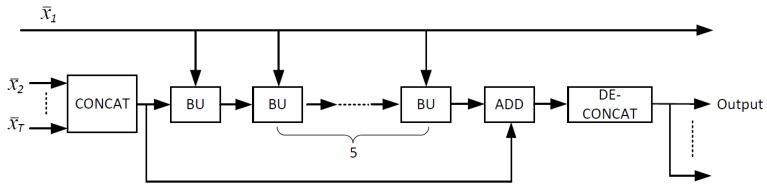
Temporal DCNN for joint reconstruction. Intermediate reconstructions ${\bar{x}}_{i=1\ldots T}$ go through this temporal DCNN together to generate the final outputs.

**Figure 5:**
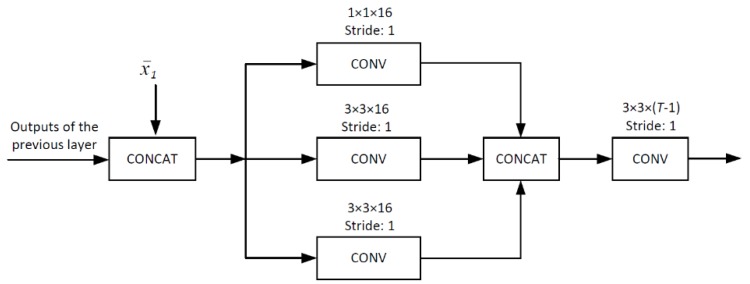
BU. The inputs are the intermediate reconstruction of key frame ${\bar{x}}_{1}$ and the outputs of the previous layer.

JsrNet utilizes the whole group of frames as the reference to reconstruct each frame, regardless of key frames and non-key frames, while the existing frameworks only utilize key frames as the reference to reconstruct non-key frames. Figure 4 shows the structure of the temporal DCNN for joint reconstruction, which is made up of several basic units (BUs). As shown in Figure 5, BU consists of a concatenating layer, an inception layer, and a convolutional layer. In the concatenating layer, we concatenate the intermediately reconstructed key frame and the output of the previous layer into a single tensor. Adaptively exploiting temporal correlation is the key to improve the overall reconstruction quality in traditional DCVS [17,18]. Therefore, we adopt the inception module [35] in the inception layer to let DCNN adaptively select the optimal size to exploit temporal correlation. In the last convolutional layer, 3 × 3 convolution kernels are utilized to reduce the number of channels from *T* to *T* − 1. ReLU activation is removed in this convolutional layer. After stacking 5 BUs, we add a shortcut connection to the plain network, making the DCNN easier to train [36]. Then, we de-concatenate the output to obtain the final reconstructed frames.

## 4. Experiments

### 4.1. Training Settings

We implemented the proposed JsrNet with Tensorflow framework using NVIDIA Titan XP GPU. UCF-101 dataset [37] was used to benchmark the proposed network because there is no standard dataset designed for DCVS. Due to limited GPU memory, we cropped the central 160 × 160 patch from each frame and retained only the luminance component. The size of group of frames was set to 4 and the batch size was set to 16. Groups were randomly split into 80% for training, 10% for validation, and the remaining for testing. The sampling rate of key frames $SR_{K}$ was set to 0.25, whereas the sampling rate of non-key frames $SR_{N}$ was set to 0.01, 0.04, and 0.1. We adopted the Adam optimizer [38] with a learning rate of 0.0001 to train JsrNet for 50 epochs.

In DCVS, the reconstruction quality of key frames plays a significant role in improving the overall reconstruction performance, because key frames are allocated with high sampling rates for guaranteed high reconstruction quality to serve as references in the recovery of non-key frames. The reconstruction quality of key frames, however, can be easily degraded by the poor reconstruction quality of non-key frames in joint optimizations. Hence, we pre-trained the sampling part and the spatial DCNN for key frames based on VOC dataset [39]. The learning rate was set to 0.0001 and the batch size was set to 128. We pre-trained the subnetwork for 200 epochs.

### 4.2. Performance Comparisons

We compared the proposed JsrNet with four state-of-the-art algorithms experimentally: (1) D-AMP [7], which is a representative of the state-of-the-art iterative algorithms developed for CS; (2) Reconnet [22], which is a dedicated DCNN-based approach for block-based CS; (3) FIR [25], which is a novel full image recovery CS framework for block-based CS; and (4) MH-BCS-SPL [11], which achieves the state-of-the-art performance in DCVS. CSVideoNet [24] is another architecture designed for DCVS and was intended to be compared; however, we could not present the results of CSVideoNet due to limited GPU memory. The parameters used in these methods were set as default to keep fairness.

**Figure 6:**
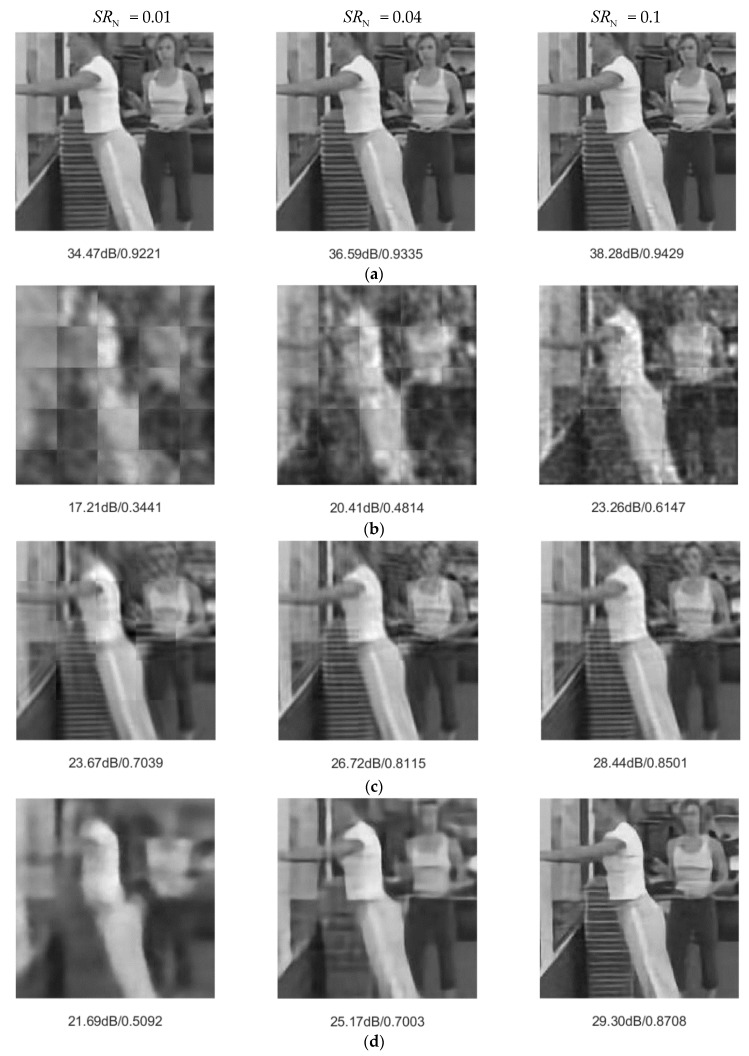

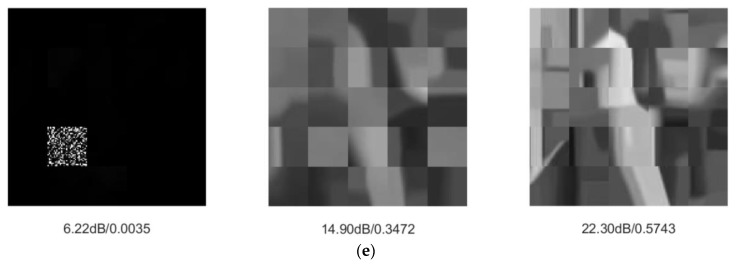
Visual comparisons of *WallPushups_g20*. (**a**) JsrNet, (**b**) Reconnet, (**c**) MH-BCS-SPL, (**d**) FIR, (**e**) D-AMP.

**Figure 7:**
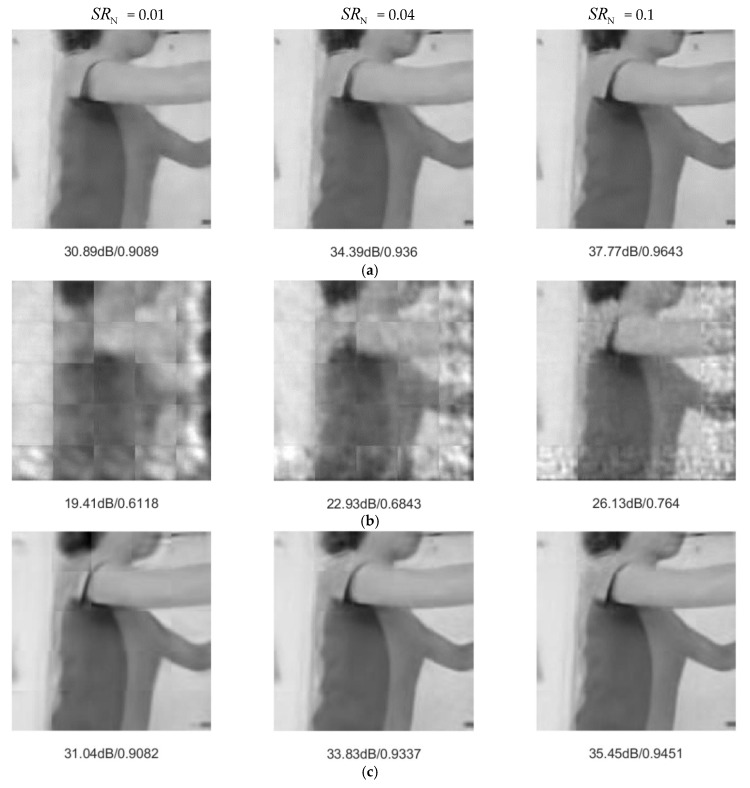

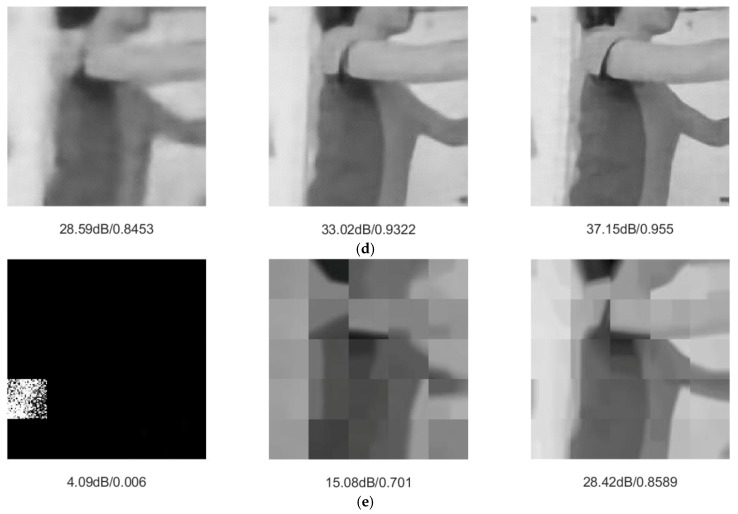
Visual comparisons of *WallPushups_g10*. (**a**) JsrNet, (**b**) Reconnet, (**c**) MH-BCS-SPL, (**d**) FIR, (**e**) D-AMP.

We adopted PSNR and structural similarity (SSIM) as objective standards to measure reconstruction performance. Table 1 shows the average PSNR and SSIM of the test set. JsrNet outperformed the other four algorithms. For example, in experiments with $SR_{N}$ = 0.01, JsrNet outperformed Reconnet, MH-BCS-SPL, FIR, and D-AMP by 8.37 dB, 2.91 dB, 4.03 dB, and 16.69 dB, respectively. Furthermore, Figure 6 and Figure 7 present examples of visual comparisons with different sampling rates. Reconnet, D-AMP, and MH-BCS-SPL suffered from blocking artifacts, especially when having low sampling rates. The main reason was that they compressed and recovered signals in a block-wise manner, but ignored edge continuity between blocks. Benefiting from exploiting temporal correlation instead of treating each frame independently, MH-BCS-SPL slightly alleviated the blocking artifacts and achieved an acceptable performance. Although FIR succeeded in reducing the blocking artifacts because all the measurements of blocks from one image were used to simultaneously reconstruct the full image, FIR failed in preserving image details. It can be seen clearly that JsrNet achieved the best performance. There were several factors contributing to this improvement. First, combining the advantages of FIR and MH-BCS-SPL, JsrNet utilized the whole group of frames as the reference to reconstruct each frame, regardless of key frames and non-key frames. JsrNet further applied the conception of exploiting complementary information between frames in joint sampling by adopting learnable convolutions to sample multiple frames jointly and simultaneously in the encoder.

Table 2 shows the comparisons of average reconstruction speed of each frame. Compared with MH-BCS-SPL and DAMP, the reconstruction time of JsrNet was nearly 1000 times faster. This was because DCNN-based approaches reconstruct video sequences via a forward model instead of solving an iterative optimization problem. More importantly, the speed of DCNN-based approaches depends only on the model capacity, whereas traditional approaches depend on the sampling rate. Compared with Reconnet and FIR, which treat each frame independently, JsrNet reconstructed frames simultaneously, and achieved the best performance.

## 5. Conclusions

A DCNN-based learning framework, named “JsrNet”, is proposed with the aim to apply DCVS in real-time applications. JsrNet utilizes the whole group of frames as the reference to reconstruct each frame, regardless of key frames and non-key frames. Moreover, JsrNet applies the conception of exploiting complementary information between frames in joint sampling by adopting learnable convolutions to sample multiple frames jointly and simultaneously in an encoder. Benefiting from fully exploiting spatial–temporal correlation in both sampling and reconstruction, JsrNet achieves a satisfying reconstruction quality without the blocking artifacts. Moreover, the non-iterative nature of DCNNs leads to low computational complexity, making JsrNet a promising candidate in source-limited, real-time scenarios. In future, we will focus on utilizing generative models for the representation and reconstruction of video sequences.

## Figures and Tables

**Table 1:** Reconstruction performance comparisons (PSNR/SSIM).

$SR_{N}$	JsrNet	Reconnet	MH-BCS-SPL	FIR	D-AMP
0.01	29.81 dB/0.8604	21.44 dB/0.5766	26.90 dB/0.7837	25.78 dB/0.7419	13.12 dB/0.2283
0.04	31.99 dB/0.9018	23.58 dB/0.6554	29.02 dB/0.8372	29.27 dB/0.8499	20.36 dB/0.6284
0.1	34.15 dB/0.9390	25.44 dB/0.7371	30.21 dB/0.8604	32.71 dB/0.9107	26.56 dB/0.7625

**Table 2:** Reconstruction speed comparisons (s).

$SR_{N}$	JsrNet	Reconnet	MH-BCS-SPL	FIR	D-AMP
0.01	0.003	0.008	4.631	0.034	14.935
0.04	0.003	0.008	3.805	0.033	14.822
0.1	0.003	0.008	1.932	0.034	13.097
